# Detection of circulating miRNAs: comparative analysis of extracellular vesicle-incorporated miRNAs and cell-free miRNAs in whole plasma of prostate cancer patients

**DOI:** 10.1186/s12885-017-3737-z

**Published:** 2017-11-09

**Authors:** Edgars Endzeliņš, Andreas Berger, Vita Melne, Cristina Bajo-Santos, Kristīne Soboļevska, Artūrs Ābols, Marta Rodriguez, Daiga Šantare, Anastasija Rudņickiha, Vilnis Lietuvietis, Alicia Llorente, Aija Linē

**Affiliations:** 1 Latvian Biomedical Research and Study Centre, Ratsupites Str 1, k-1, Riga, LV-1067 Latvia; 2 Riga Stradiņš University, Dzirciema Str 16, Riga, LV-1007 Latvia; 3 Department of Molecular Cell Biology, Institute for Cancer Research, Oslo University Hospital-The Norwegian Radium Hospital, 0379 Oslo, Norway; 4 Institute of Clinical and Preventive Medicine, Faculty of Medicine, University of Latvia, Raina blvd. 19, Riga, LV – 1586 Latvia

**Keywords:** Prostate cancer, Cell-free miRNAs, Extracellular vesicles, Exosomes, Microvesicles, Biomarkers, Liquid biopsy

## Abstract

**Background:**

Circulating cell-free miRNAs have emerged as promising minimally-invasive biomarkers for early detection, prognosis and monitoring of cancer. They can exist in the bloodstream incorporated into extracellular vesicles (EVs) and ribonucleoprotein complexes. However, it is still debated if EVs contain biologically meaningful amounts of miRNAs and may provide a better source of miRNA biomarkers than whole plasma. The aim of this study was to systematically compare the diagnostic potential of prostate cancer-associated miRNAs in whole plasma and in plasma EVs.

**Methods:**

RNA was isolated from whole plasma and plasma EV samples from a well characterised cohort of 50 patient with prostate cancer (PC) and 22 patients with benign prostatic hyperplasia (BPH). Nine miRNAs known to have a diagnostic potential for PC in cell-free blood were quantified by RT-qPCR and the relative quantities were compared between patients with PC and BPH and between PC patients with Gleason score ≥ 8 and ≤6.

**Results:**

Only a small fraction of the total cell-free miRNA was recovered from the plasma EVs, however the EV-incorporated and whole plasma cell-free miRNA profiles were clearly different. Four of the miRNAs analysed showed a diagnostic potential in our patient cohort. MiR-375 could differentiate between PC and BPH patients when analysed in the whole plasma, while miR-200c-3p and miR-21-5p performed better when analysed in plasma EVs. EV-incorporated but not whole plasma Let-7a-5p level could distinguish PC patients with Gleason score ≥ 8 vs ≤6.

**Conclusions:**

This study demonstrates that for some miRNA biomarkers EVs provide a more consistent source of RNA than whole plasma, while other miRNAs show better diagnostic performance when tested in the whole plasma.

## Background

Circulating cell-free micro-RNAs (miRNAs) have emerged as promising biomarkers for the development of blood-based assays for early detection, prognosis and monitoring of cancer. In 2008, Mitchell et al. demonstrated for the first time that miRNAs are released from prostate cancer (PC) cells into the bloodstream, where they exist in a remarkably stable form [1]. miRNAs were shown to remain stable after incubation of plasma or serum at room temperature for up to 24 h and to resist RNase A digestion, HCl and NaOH treatment or multiple freeze-thaw cycles [1, 2]. Subsequently, the levels of circulating miRNAs have been studied in patients with various cancers, including PC, resulting in the discovery of individual miRNAs or miRNA signatures with diagnostic and/or prognostic value [3].

PC is the most frequently diagnosed cancer in males in Europe and the United States [4, 5]. Currently, the serum PSA test is the most commonly used tool for organised screening programs, opportunistic screening and monitoring of PC [6]. However, PSA is not cancer specific and the high false-positive rate and low specificity leads to large numbers of unnecessary prostate biopsies and emotional morbidity [7]. Furthermore, PC is characterised by a highly heterogeneous course - one part of the patients develops a high-grade disease with extracapsular spread and distant metastases requiring aggressive treatment, while others have a relatively indolent, slowly progressing disease that could have been managed by active surveillance [8]. The current standard of care analyses, however, do not predict whether a histologically proven tumour will give rise to a clinically significant disease, leading to overtreatment of indolent PC. Hence, the greatest unmet clinical needs in the management of PC are sensitive and reliable non-invasive tools for differentiating between PC and benign prostatic diseases, and between potentially fast progressing PC requiring aggressive treatment and a relatively indolent disease that can be managed by active surveillance.

More than 20 studies investigating levels of cell-free miRNAs in plasma or serum of PC patients have been published up to date [9, 10]. The majority of these studies were focused on the identification of circulating miRNAs that differentiate between patients with PC and benign prostatic hyperplasia (BPH) or healthy controls. Some of these studies have shown remarkably high diagnostic value. For example, Chen et al. identified a 5 miRNA panel that could differentiate PC from BPH with an AUC of 0.924 and PC from healthy controls with an AUC of 0.860 [11]. Some other studies have reported cell-free miRNAs that differentiate between localised and metastatic castration resistant prostate cancer (mCRPC) or between low-grade and high-grade PC. For example, Mihelich et al. developed a “miRScore” that based on the serum levels of 14 miRNAs could predict absence of high-grade PC among men with PC and BPH with a negative predictive value of 0.939 [12]. However, relatively few miRNA biomarkers have been validated by several independent studies, while many other miRNAs either have been reported in a single study or show conflicting results [3, 10]. Therefore, the analysis of cell-free miRNAs is regarded as a poorly reproducible technique [3, 13, 14].

Cell-free miRNAs circulating in the bloodstream have been found to be enclosed into extracellular vesicles (EVs) [15, 16], or to exist in a vesicle-free form associated with high-density lipoproteins [17], Ago2 protein [18, 19] or other RNA binding proteins [20]. The majority of the studies has used whole plasma or serum as a source of cell-free miRNAs. However, it has recently been hypothesised that cancer-derived EVs may be enriched with miRNA signatures reminiscent of their cell of origin, contain rare yet highly specific RNA biomarkers and protect their RNA cargo from degradation in the bloodstream and, therefore, the analysis of EV-enclosed miRNAs may be superior to whole plasma/serum analysis [10, 21, 22]. Nevertheless, to the best of our knowledge, a direct comparison of miRNA detection assays in whole plasma and plasma EVs has not been reported so far.

In this study, we evaluated the performance of 9 miRNA biomarkers previously reported to have a diagnostic or prognostic significance in PC by quantifying them in the whole plasma and plasma EVs in a cohort of 50 PC and 22 BPH patients.

## Methods

### Study population and sample collection

Patients with PC and BPH were recruited between September 2011 and December 2013 at Riga East University Hospital and subsequently were followed up until December 2016. The diagnosis was established using standard of care diagnostic examinations and Gleason score was determined according to standard histopathological criteria by an experienced pathologist.

Pre-treatment blood samples were collected into EDTA-coated tubes and processed at room temperature within 2 h of blood draw. Plasma samples were centrifuged twice for 10 min at 2000 *g*, aliquoted and stored at −80 °C until analysis. The samples were deposited into the Latvian Genome Database. Biobanking procedures were approved by the Committee of Medical Ethics of Latvia and the use of clinical samples for the research was approved by the Committee of Biomedical Ethics of Riga East University Hospital. The blood samples were collected after the patients’ informed written consent was obtained.

The following groups of patients were selected from the Database: PC with Gleason score ≥ 8 (Gleason high, *n* = 24), PC with Gleason score ≤6 (Gleason low, *n* = 26) and BPH (absence of PC confirmed by histological examination of ultrasound-guided needle biopsies and no change in the diagnosis within the follow-up period, *n* = 22). Clinical data of the study population are provided in Table 1. In addition, plasma samples from 5 PC patients and 5 healthy controls were used for the quality control of EV isolation.

**Table 1:** Clinical characteristics of the study population

Characteristics	Prostate cancer, *n* = 50	Benign prostatic hyperplasia, *n* = 22
Age (years)		
Mean ± SD	66 ± 7	61 ± 8
Median (range)	65 (54–85)	60 (44–75)
Missing	1	4
Serum PSA (ng/ml)		
0–4.0	3 (6%)	9 (41%)
4.1–20.0	31 (62%)	13 (59%)
> 20.0	15 (30%)	0 (0%)
Missing	1 (2%)	0 (0%)
Gleason score		
4–6	26 (52%)	–
8–9	24 (48%)	–
Metastasis status		
M0	39 (78%)	–
M1	3 (6%)	–
Missing	8 (16%)	–
Cancer grade		
G1	0 (0%)	–
G2	11 (22%)	–
G3	12 (24%)	–
Missing	27 (54%)	–
Prostatitis		
–	40 (80%)	11 (50%)
+	8 (16%)	11 (50%)
Missing	2 (4%)	0 (0%)

### Isolation of extracellular vesicles

EVs were isolated from 400 μl of plasma using size exclusion chromatography (SEC). SEC columns were prepared by filling TELOS SPE columns (Kinesis, USA) with 10 ml (bed volume) of CL6B sepharose (GE Healthcare, USA). Plasma samples were loaded on the columns and gravity-eluted with PBS. The eluate was collected in 12 sequential 0.5 ml fractions. Each fraction was measured by Zetasizer Nano ZS (Malvern, UK) and fractions containing particles larger than 30 nm were combined and concentrated to 100 μl using Amicon Ultra 3 kDa centrifugal filters (Merck, Millipore, Germany).

### Transmission electron microscopy

Ten μl of EV suspension in PBS were applied to 300-mesh carbon coated copper EM grid and incubated for 5 min. Then the samples were negatively stained with 1% uranyl formate (*w*/*v*) for 1 min, dried and examined using JEM-1230 transmission electron microscope (JEOL, USA).

### Nanoparticle tracking analysis

Size distribution profile and concentration of EVs was determined using NanoSight NS500 instrument (Malvern, UK). EV samples were diluted 1000–25,000 fold in PBS to achieve particle concentration in range from 1×10^8^ to 1×10^9^ particles/ml. For each sample, five 30 s videos were recorded with the following settings: 25C, 0.944–0.948 cP, 1259 slider shutter, 366 slider gain, and 11 camera level. The data analysis was performed with NanoSight NTA Software v3.1 Build 3.1.54 in the auto mode.

### Western blot

EVs and PC-3 cells (used as a positive control) were lysed in RIPA buffer (150 ml NaCl, 1% Triton X-100, 0.5% Na deoxycholate, 0.1% SDS, 50 ml Tris) and the protein concentration was assessed using Pierce™ BCA Protein Assay Kit (Thermo Fisher Scientific, USA) following manufacturer’s instructions. Thirty micrograms of EV and cell proteins were mixed with Laemmli buffer under reduction conditions, denatured for 5 min at 100 °C and loaded on 10% SDS-PAGE gel. Proteins were electroblotted to nitrocellulose membranes and the membranes were blocked with 10% (*w*/*v*) fat-free milk and then incubated with the following primary antibodies: anti-TSG101 (Abcam, # ab125011), Calnexin (Abcam, # ab22595), CD9 (Santa Cruz Biotechnology, # sc-13118) and β-actin (Abcam, # ab8224) in 1:1000 dilution. The blots were washed and incubated with horseradish peroxidase-conjugated goat anti-rabbit IgG F(ab’)2-HRP (1:2000) (Santa Cruz, #sc-3837) or chicken anti-mouse IgG-HRP (1:2000) (Santa Cruz, #sc-2962) secondary antibodies, respectively. Protein expression was visualized using Western Blotting Detection Reagent kit (GE HealthCare Lifesciences, Germany).

### Enzymatic treatment

Prior to RNA extraction, EVs samples were treated with 1 mg/ml proteinase K (Thermo Fisher Scientific, USA) for 30 min at 37 °C. Proteinase K was inactivated by incubating the samples for 10 min at 65 °C. Then the samples were treated with 10 ng/μl RNase A (Thermo Fisher Scientific, USA) for 15 min at 37 °C.

### RNA extraction

RNA was extracted from EV and whole plasma samples using miRNeasy Micro Kit (Qiagen, USA) according to the manufacturer’s instructions with slight modifications of the protocol. Briefly, 5 volumes of QIAzol Lysis Reagent were added to each sample. Subsequently, samples were spiked with 1 μl of UniSp6 (Exiqon, Denmark), which was used as a normaliser in downstream analysis. After adding 1 volume of chloroform samples were centrifuged for 15 min at 12000 *g* at 4 °C and the aqueous phase was transferred to a new tube. Then, 1.5 volumes of 100% ethanol were added to each sample and the mixture was loaded onto a MinElute spin column. Columns were centrifuged at 1000 *g* for 30 s at room temperature in each round until entire sample was loaded. RNA was eluted in 15 μl of RNase-free water using low-bind tubes. The quantity and quality of RNA was assessed using Agilent 2100 Bioanalyzer and RNA 6000 Pico Kit (Agilent technologies, # 5067–1513).

### RT-qPCR analysis

One third of each RNA sample isolated from EVs and whole plasma was reverse-transcribed using miRCURY LNA Universal cDNA Synthesis kit II (Exiqon) according to the manufacturer’s protocol. cDNA reaction mixtures were diluted 1:40 and 4 μl were used for qPCR reactions. qPCR was carried out using microRNA LNA PCR primer sets and ExiLENT SYBR Green master mix (Exiqon) according to the manufacturer’s protocol on ViiA 7 Real-Time PCR system (Thermo Fisher Scientific).

### Statistical analysis

Ct values were averaged between duplicates and normalized against UniSp6 spike-ins by subtracting them from average spike-in Ct values in the same samples, resulting in log2 relative quantities (log2 RQ’s). The statistical analyses were performed with GraphPadPrism 5 (GraphPad, USA). A non-parametric Mann-Whitney *U* test was used to compare the RQ values of each miRNA between the groups of samples. Multiple testing correction was done by false discovery rate (FDR) estimation and adjusted (adj.) *P*-value of ≤0.05 was considered to be significant. To assess the diagnostic potential, the area under the ROC curve (AUC) was calculated for each miRNA.

## Results

### Selection of miRNA biomarkers

Nine miRNAs, whose levels in plasma or serum have been reported to have a diagnostic or prognostic significance in PC in at least two independent studies, were selected for this study. Studies showing their relevance for the diagnosis or prognosis of PC are summarised in Table 2. MiR-21-5p, miR-200c-3p, miR-210-3p and miR-375 have been shown to be increased in the blood of PC patients as compared to BPH or healthy controls consistently by two or more studies, while miR-30c-5p and miR-223-3p were found to be consistently decreased in the blood of PC patients. Inconsistent findings have been reported for Let-7a-5p, miR-141-3p and miR-106a-5p.

**Table 2:** Circulating cell-free miRNA biomarkers for prostate cancer

miRNA	Expression in PC tissues		Level in blood				
	Direction	Ref.	Sample type	Patient groups and sample size	Direction	Normalisation	Ref.
Let-7a-5p	Down in PC vs adj. Normal tissues	[45]	Serum	PC (n = 75), BPH (*n* = 27)	Down in PC	RNA input and miR-16, miR-425	[52]
	Down in PC vs BPH	[44]	Serum	High grade PC (n = 50), low grade PC (n = 50), BPH (n = 50)	Down in high grade PC vs low grade PC, BPH	RNA input and spike-ins	[12]
			Serum	Disseminated PC (*n* = 20), BPH (n = 13)	Up in disseminated PC	Spike-in and miR-320a	[37]
miR-21-5p	Up in PC vs adj. Normal (*n* = 10)	[55]	Plasma	mCRPC (*n* = 25, pooled), LPC (*n* = 25, pooled)	Up in mCRPC	miR-30e	[40]
	Similar in PC and adj. Normal tissues (*n* = 36)	[56]	Serum	ADPC (*n* = 20), HRPC (*n* = 10), LPC (n = 20), BPH (*n* = 6)	Up in HRPC vs ADPC, LPC	U6 snRNA	[42]
	Up in PC vs normal tissues	[57]	Plasma	PC (*n* = 51), HC (n = 20)	Up in PC	RNU1A snRNA	[43]
miR-30c-5p	Up in PC vs adj. Normal epithelium (*n* = 37)	[58]	Serum	High grade PC (n = 50), low grade PC (n = 50), BPH (n = 50)	Down in high grade PC vs low grade, BPH	RNA input and spike-ins	[12]
	Up in PC vs normal tissues	[57]	Plasma	PC (*n* = 80), BPH (*n* = 44), HC (*n* = 54)	Down in PC vs BPH, HC	U6 snRNA	[11]
			Serum	PC (n = 36), HC (n = 12)	Down in PC	RNA input	[51]
miR-106a-5p	Up in PC vs normal tissues	[57]	Serum	High grade PC (n = 50), low grade PC (n = 50), BPH (n = 50)	Down in high grade PC	RNA input and spike-ins	[12]
			Serum	PC (*n* = 36), HC (*n* = 12)	Up in PC	RNA input	[51]
miR-141-3p	Up in mPC, PC vs normal tissues	[53]	Serum	High grade PC (n = 50), low grade PC (n = 50), BPH (n = 50)	Detectable in <50% of patients	RNA input and spike-ins	[12]
	Up in PC vs BPH	[52]	Serum	PC (*n* = 75), BPH (*n* = 27)	Up in PC	RNA input and miR-16, miR-425	[52]
	Up in BCR after RP vs. no BCR after RP	[59]	Plasma	mCRPC (*n* = 25, pooled), LPC (n = 25, pooled)	Up in mCRPC	miR-30e	[40]
			Serum	mCRPC (n = 26), low-risk LPC (n = 28)	Up in mCRCP	U6 snRNA	[53]
	Up in PC (n = 36) vs normal tissue (n = 36)	[54]	Plasma EVs	PC (*n* = 78), HC (*n* = 28)	Up in PC	Spike-ins	[38]
			Serum EVs	mPC (n = 47), non-recurrent PC (*n* = 72)	Up in mPC		
			Serum	71 PC: N1 (*n* = 48), N0 (*n* = 23), GS ≥8 (*n* = 29), GS = 7 (*n* = 42)	Up in N1 PC vs N0 PC; Up in GS ≥ 8 vs GS = 7	Spike-ins	[54]
			Plasma	mPC (n = 25), LPC (*n* = 26)	Up in mPC vs LPC; Similar in PC and HC	RNU1A snRNA	[43]
			Serum	mPC (n = 25), HC (n = 25)	Up in mPC	Spike-ins	[60]
			Serum	PC (n = 54), non-malignant (*n* = 79)	Up in higher GS; Similar in PC and non-malignant	RNU1–4 and SNORD43	[61]
miR-200c-3p	Up in PC vs normal tissue	[62]	Plasma	mCRPC (n = 25, pooled), LPC (n = 25, pooled)	Up in mCRPC	miR-30e	[40]
			Serum	mCRPC (n = 25), HC (n = 25)	Up in mCRCP	Spike-ins	[41]
miR-210-3p	Up in PC vs BPH	[44]	Serum	PC (*n* = 31), BPH (*n* = 13)	Up in PC	Spike-in and miR-320a	[37]
			Serum	mCRPC (*n* = 21), HC (n = 20)	Up in mCRCP	Spike-ins	[41]
miR-223-3p	Up in PC vs adj. Normal tissues (n = 10)	[63]	Serum	High grade PC (n = 50), low grade PC (n = 50), BPH (n = 50)	Down in high grade PC vs low grade, BPH	RNA input and spike-ins	[12]
	Up in PC vs normal tissues	[57]	Serum	PC (n = 36), HC (n = 12)	Down in PC	RNA input	[51]
miR-375	Up in mPC, PC vs normal tissues	[53]	Plasma EVs	CRPC (*n* = 100)	High miRNA level associated with poor OS	RNA input and miR-30a-5p, miR-30e-5p	[39]
			Serum	PC (*n* = 31), BPH (n = 13)	Up in PC	Spike-in and miR-320a	[37]
	Up in PC (n = 36) vs normal tissue (n = 36)	[54]	Plasma	mCRPC (n = 25, pooled), LPC (n = 25, pooled)	Up in mCRPC	miR-30e	[40]
			Serum	mCRPC (n = 26), low-risk LPC (n = 28)	Up in mCRCP	U6 snRNA	[53]
			Serum EVs	mPC after RP (*n* = 47), non-recurrent PC after RP (*n* = 72)	Up in mPC	Spike-ins	[38]
			Serum	71 PC: N1 (n = 48), N0 (*n* = 23), GS ≥8 (*n* = 29), GS = 7 (*n* = 42)	Up in N1 PC vs N0 PC; similar in GS ≥ 8 and GS = 7	Spike-ins	[54]

*ADPC* androgen-dependent prostate cancer, *BCR* biochemical recurrence, *BPH* benign prostatic hyperplasia, *CRPC* castration resistant prostate cancer, *EVs* extracellular vesicles, *HC* healthy control, *HRPC* hormone-refractory prostate cancer, *LPC* localized prostate cancer, *mCRPC* metastatic castration resistant prostate cancer, *mPC* metastatic prostate cancer, *PC* prostate cancer, *RP* radical prostatectomy

### Yield and purity of EVs

**Fig. 1:**
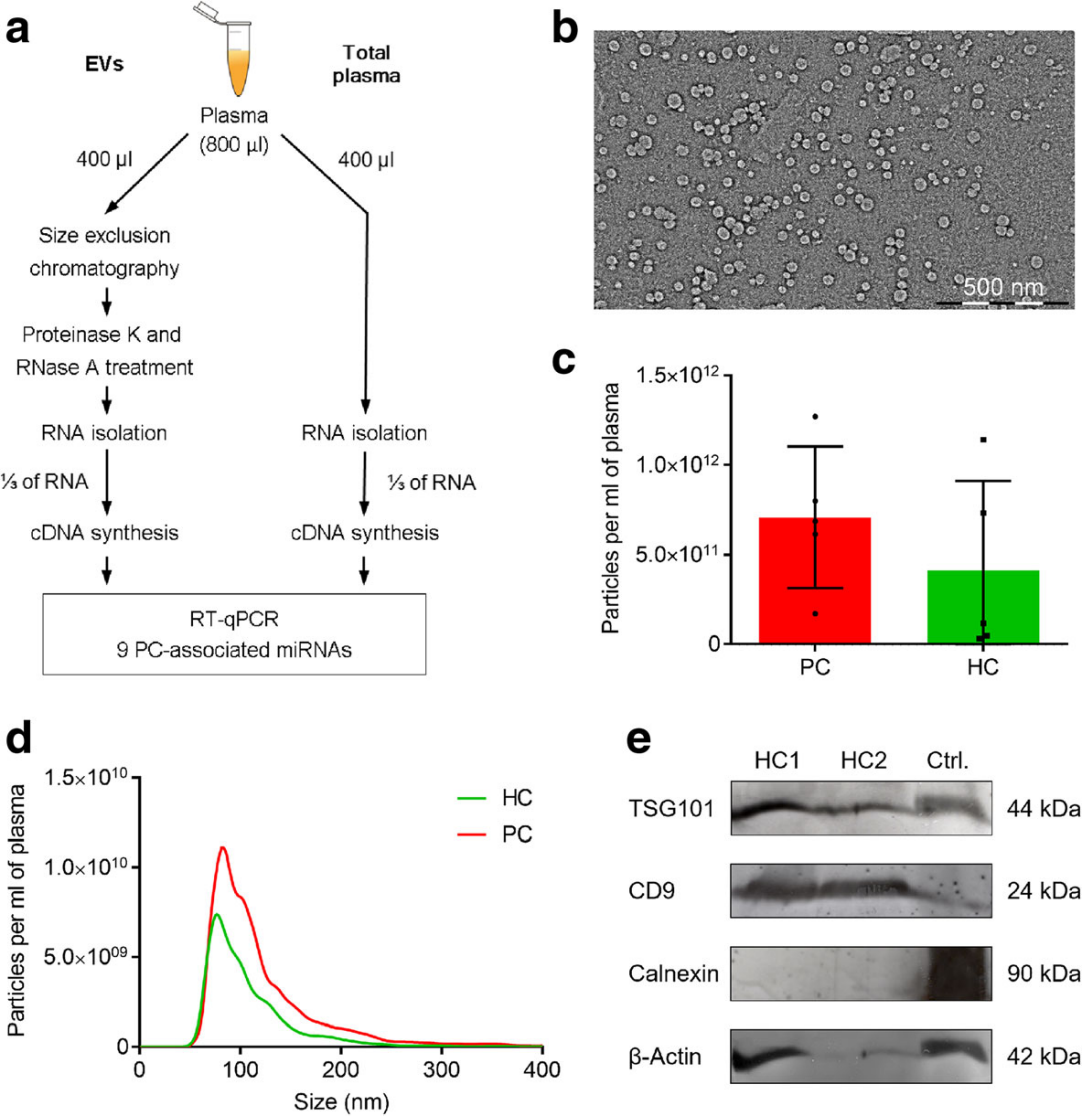
Workflow of the study and characterisation of plasma EVs. **a** Workflow of the study. **b** Representative transmission electron microscopy image of plasma EVs. **c** Quantification of EVs isolated from plasma of PC patients and healthy controls (HC) by nanoparticle tracking analysis. **d** Average size distribution of EVs isolated from plasma of PC patients and healthy controls. **e** Western blot analysis of EV markers (TSG101, CD9), endoplasmic reticulum protein Calnexin and β-actin in plasma EVs isolated from two healthy individuals and PC-3 cells (as a positive control)

In order to compare the levels of the selected miRNAs in plasma EVs and whole plasma, each plasma sample was divided into two 400 μl aliquots – one was used for the isolation of EV-incorporated RNA, while another was used directly for the isolation of cell-free RNA from whole plasma according to the workflow shown in Fig. 1a.

To assess the yield and purity of EVs, EV samples from 5 PC patients and 5 healthy controls (not included in the miRNA analysis) were characterised by transmission electron microscopy (TEM), nanoparticle tracking analysis (NTA) and Western blot analysis. TEM images revealed that the majority of particles were ranging in size from 25 to 60 nm that corresponds to the size of exosomes (Fig. 1b). However, as it has been shown that SEC-based EV isolation methods do not result in lipoprotein-free EV preparations [23], it cannot be excluded that a fraction of the particles are lipoproteins. NTA showed that the concentrations of EVs range from 3.14×10^10^ to 1.27×10^12^ particles per ml of plasma (Fig. 1c). The EV count was slightly increased in plasma from PC patients as compared to the healthy controls (mean count in PC 7.08×10^11^ vs 4.15×10^11^ in healthy controls), although the difference didn’t reach statistical significance in our sample set. The size distribution analysis showed that the diameter for the majority of particles was in the range from 50 to 150 nm with a minor fraction reaching ~230 nm (Fig. 1d), which is somewhat inconsistent with the TEM results. This discrepancy likely has arisen due to the difference in the minimum detectable EV size between both techniques [24] and /or shrinking of EVs during fixation for TEM [25]. Western blot analysis showed that the EVs were positive for typical EV markers TSG101 and CD9, and negative for the endoplasmic reticulum protein Calnexin (Fig. 1e). Taken together, these results show that the EV isolation method used in this study results in a relatively high yield of exosome-enriched EV preparations without detectable contamination of intracellular components.

### RNA profiles in EVs and whole plasma

**Fig. 2:**
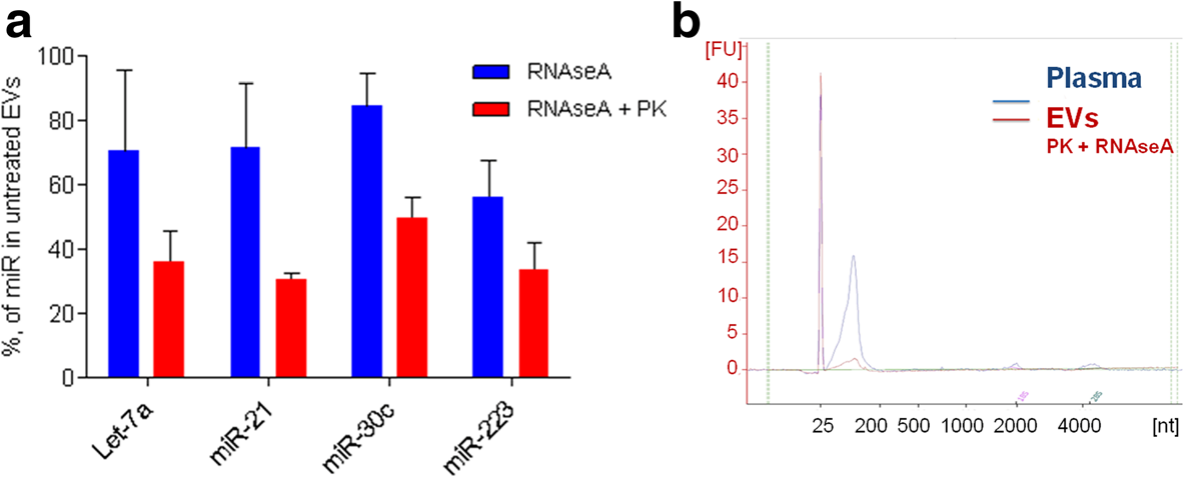
Effects of proteinase K and RNase A treatment on the relative quantity of EV-incorporated miRNAs and RNA profiles in whole plasma and EVs. **a** RT-qPCR analysis of miRNA levels in EVs treated with RNase A alone or with a combination of proteinase K and RNase A relatively to untreated EVs. Bars show the mean percentage in EVs from 3 healthy individuals. **b** A representative RNA profile from whole plasma and EVs treated with proteinase K and RNase A obtained by Bioanlyzer RNA 6000 Pico chip

As it has been suggested that EVs may associate with lipoproteins or protein complexes that carry cell-free miRNAs and protect them from degradation [18, 26], we first tested the effect of proteinase K and RNase A treatment on the miRNA levels in plasma EVs from three healthy individuals (Fig. 2a). Treatment of EVs with RNase A alone reduced the relative quantity (RQ) values by 15.5 to 43.6% for different miRNAs, while the treatment with proteinase K prior to RNase A resulted in the reduction of RQs by 50.4 to 69.3%. This suggests that the proteinase K treatment is required for efficient removal of extra-vesicular RNA. Therefore, in order to study the intraluminal miRNAs, all EV preparations were treated with proteinase K and RNase A prior to the RNA extraction. RNA was extracted from EVs and whole plasma using miRNeasy Micro kit, which is designed for isolation of total RNA from small amounts of sample. Typical RNA profiles obtained by Bioanalyzer from whole plasma and EVs are shown in Fig. 2b. The profiles show the presence of small RNA peaks of 25 to 200 nt both in whole plasma and EVs, while 18S and 28S rRNA peaks are present in whole plasma and EVs without the enzymatic treatment (not shown) but not in the treated EVs, thus suggesting that the majority of rRNA is bound to the surface of EVs.

### Relative abundance of EV-incorporated miRNAs

**Fig. 3:**
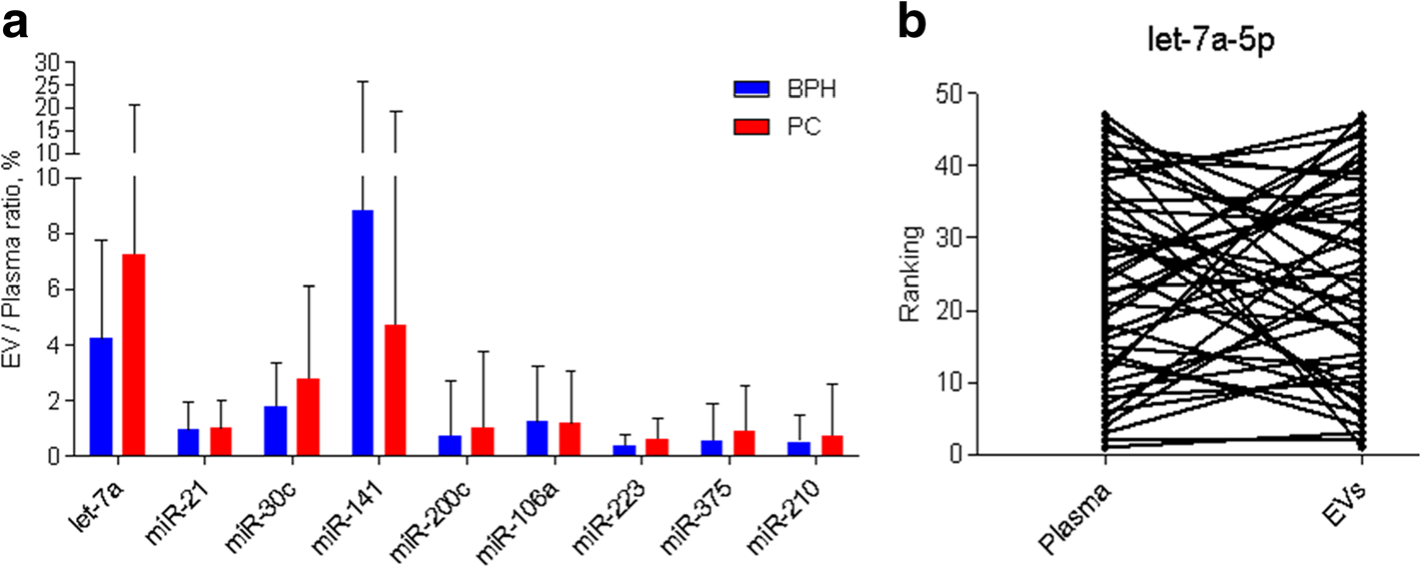
Relative abundance of EV-incorporated miRNAs. **a** Ratio between EV-incorporated and total cell-free miRNAs in whole plasma. Bars represent the mean ratios in groups of patients with PC and BPH. **b** A paired dot plot shows the ranking of PC patients according to Let-7a-5p levels in EVs and whole plasma; lines connect the samples from the same individual

An equal proportion (one third) from the total RNA amount obtained from the EV and whole plasma samples of PC and BPH patients was used for the RT-qPCR analysis of the 9 selected miRNA biomarkers. Spike-ins were used to control for a variation in RNA extraction, cDNA synthesis and PCR efficiency and they typically varied less than by 1 Ct. In order to assess the relative abundance of EV-enclosed miRNAs, a ratio between EV-enclosed and total cell-free miRNAs in whole plasma was calculated (Fig. 3a). The results showed that only a small fraction of the cell-free miRNA was retrieved from the EVs. However, the EV-enclosed fraction was not uniformly low – it varied from 6.36% for Let-7a-5p to 0.65% for miR-210-3p. Spearman correlation analysis revealed only weak to moderate correlation between EV-enclosed and whole plasma cell-free miRNAs (Table 3). As an example, a paired dot plot in Fig. 3b shows the discordance in the Let-7a-5p levels in EVs and whole plasma from the same patients. These data support the idea that EV-enclosed miRNA profile differs from cell-free miRNA profile in the whole plasma.

**Table 3:** Spearman correlation coefficients of EV-enclosed and whole plasma miRNAs

miRNA	Spearman r	95% confidence interval	*p* value
miR-375	0.37	0.15–0.56	0.0013
miR-141-3p	0.36	0.13–0.55	0.0018
miR-200c-30	0.37	0.13–0.56	0.0023
miR-21-5p	0.50	0.28–0.66	<0.0001
miR-30c-5p	0.42	0.19–0.60	0.0005
miR-106a-5p	0.37	0.13–0.57	0.0021
miR-223-3p	0.57	0.37–0.72	<0.0001
Let-7a-5p	0.27	0.02–0.48	0.03
miR-210-3p	0.28	0.05–0.049	0.01

Clearly, the size of the EV-enclosed miRNA fraction depends on the efficacy of the EV isolation method and the obtained ratios are not expected to represent the EV-enclosed: EV-free miRNA ratio. However, the NTA data showed that the EV count recovered in this study was similar or even higher than that reported by other studies [27–30], therefore we assume that the EV yield in our study is representative of that obtained by the current standard EV isolation techniques.

These results show that although only a small fraction of the total cell-free miRNA present in plasma was recovered from EVs, the EV-incorporated miRNA profile is clearly different from that in the whole plasma.

### Diagnostic potential of EV-enclosed and total cell-free miRNAs

**Fig. 4:**
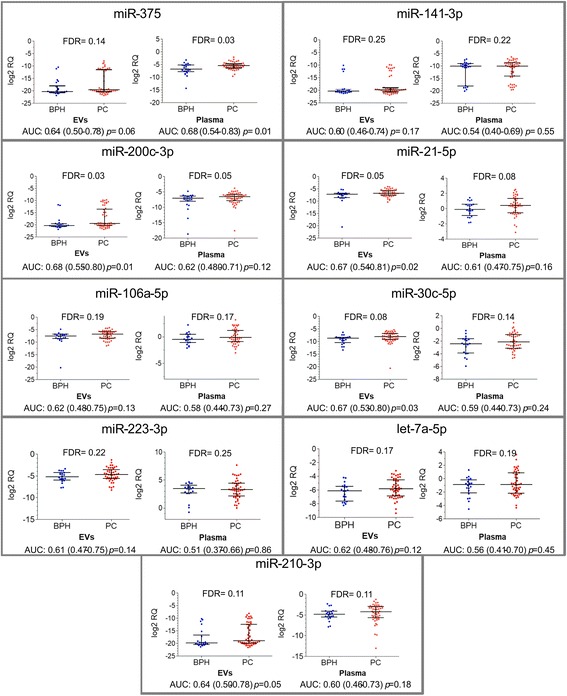
Circulating miRNA levels in patients with BPH and PC. Scatter plots show the log2RQ values of each miRNA tested in EVs and in whole plasma. FDR-adjusted *p* values are show at the top of each graph. Area under the ROC curve (AUC), 95% confidence interval and *p* value for differentiating between PC and BPH is shown below each graph

To assess the diagnostic potential of the selected miRNAs, their relative quantity in EVs and whole plasma was compared between patients with PC (*n* = 50) and BPH (*n* = 22). Three of the 9 miRNAs tested showed a diagnostic value in our sample set (Fig. 4). MiR-375 was significantly increased in PC patients as compared to BPH (FDR adj. *p* = 0.03) and had an AUC of 0.68 (95% CI: 0.54–0.83, *p* = 0.01), when tested in the whole plasma. The same tendency was observed for EV-enclosed miR-375, however it didn’t reach statistical significance. On the contrary, miR-200c-3p and miR-21-5p could differentiate between PC and BPH better when tested in EVs than in the whole plasma (AUC of 0.68, *p* = 0.01 and 0.67, *p* = 0.02, respectively, when tested in EVs and AUC of 0.62, *p* = 0.12 and AUC of 0.61, *p* = 0.16, respectively, when tested in whole plasma). The levels of the other miRNAs were not significantly different in PC samples compared to BPH neither in EVs nor in whole plasma.

**Fig. 5:**
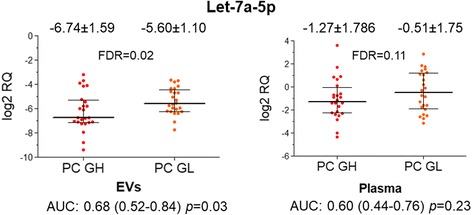
Circulating Let-7a-5p levels in PC patients with low and high Gleason score. Scatter plots show the log2RQ values of Let-7a-5p tested in EVs and in whole plasma of patients with Gleason score ≥ 8 (PC GH) and Gleason score ≤6 (PC GL). The mean log2RQ values and standard deviation is shown above each scatter plot. Area under the ROC curve (AUC), 95% confidence interval and *p* value for differentiating between PC patients with high and low Gleason score is shown below each graph

Next, we investigated the association of EV-enclosed and whole plasma miRNA levels with PC aggressiveness. We found that the level of Let-7a-5p was significantly decreased in EVs from PC patients with high Gleason score (≥8) compared to low Gleason score (≤6) and it could differentiate between these groups with AUC of 0.68 (95% CI: 0.52–0.84, *p* = 0.03) (Fig. 5). Although the same tendency was observed in whole plasma, the standard deviation was larger and statistical significance was not reached. No other miRNA could differentiate between PC patients with high and low Gleason scores.

Finally, none of the miRNAs was associated with the presence of histologically confirmed prostatitis in PC and BPH patients, thus showing that the alterations in the miRNA levels are not due to prostatic inflammation.

## Discussion

Cells can release miRNAs to the extracellular space either incorporated into EVs [31, 32] or in a vesicle-free form bound to various protein and lipoprotein complexes [17–20]. Quantification of these miRNAs in blood from cancer patients may offer new opportunities for diagnosis, prognosis, monitoring of treatment response and early detection of recurrence in a minimally invasive way. However, human blood contains a complex mixture of miRNAs derived from various cell types and, therefore, robust quantification of cancer-derived cell-free miRNAs has turned out to be a challenging task [14]. Currently, it is still debated if the EV-based miRNA detection assays are superior to the whole plasma-based assays. miRNA profiles in cancer-derived EVs have been found to be reminiscent of their cell-of-origin [31, 33], though due to selective RNA sorting mechanisms they may be enriched or depleted of some specific miRNAs [34]. The EV membrane protects the RNA cargo from degradation in the bloodstream and the intraluminal RNA content is thought to be relatively stable, therefore EVs may provide a more consistent source of miRNA biomarkers than whole plasma [15, 30]. On the other hand, it has been calculated that there is far less than one molecule of a given miRNA per EV [35], which raises the question of whether all EVs contain miRNAs and if the amounts are biologically meaningful. Moreover, it can be argued that the EV isolation step may introduce a higher variation and result in a low RNA yield that in turn would lead to lower sensitivity, higher standard deviations and poor reproducibility of the EV-based miRNA assays as compared to whole plasma assays.

Here, we have performed a systematic comparison of miRNA levels in whole plasma and EVs isolated from the same plasma samples in a well-characterised cohort of PC and BPH patients. Our results show that EV-incorporated miRNA constitutes only a minor fraction of whole plasma miRNA. This is in line with a study by Chevillet et al. showing that exosome fractions contained a small minority of the miRNA content of plasma [35]. Nevertheless, the miRNA levels in EVs and whole plasma were poorly correlated, and the EV-incorporated and whole plasma miRNA profile was clearly different. This finding is consistent with a NGS-based study by Cheng et al. that compared small RNA profiles in EVs, plasma and serum of 3 healthy individuals and showed that the miRNA levels differ remarkably between plasma and serum EVs and between EVs and cell-free plasma and serum [30].

Three out of 9 miRNAs analysed could differentiate between PC and BPH patients in our cohort. MiR-375 showed a better diagnostic performance when tested in whole plasma as compared to EVs. MiR-375 is an oncogenic miRNA that is overexpressed in tumours with high Gleason score and more advanced pathological stage [36]. Increased plasma or serum levels of miR-375 in patients with PC vs BPH or metastatic CRPC vs localised PC have been reported before in several studies (Table 2), and the AUC obtained in our study was similar to that reported before [37]. MiR-375 had one of the lowest EV to whole plasma ratios among the miRNAs analysed in this study and it was undetectable in a significant portion of EV samples. It still may have diagnostic properties in cases where it is detectable, though proving its diagnostic value would require a larger cohort of samples. Two studies have reported the presence of miR-375 in blood EVs from PC patients. Bryant et al. showed that its level is increased in serum EVs from patients with metastatic PC as compared to non-recurring PC [38], and Huang et al. reported that high EV-miR-375 level is associated with a poor prognosis in CRPC [39]. Hence, increased levels of EV-incorporated miR-375 appear to be associated with metastatic disease. As only 3 of the patients in our cohort had a metastatic disease at the time of the blood draw, we reasonably detected it only in a minority of PC patients in our cohort. Moreover, as these studies did not describe treatment of EVs with proteinase K, it is possible that the EV preparations also contained protein-bound miRNAs co-isolated with EVs.

On the contrary, EV-incorporated miR-200c-3p and miR-21-5p showed better diagnostic performance than in whole plasma. Increased plasma or serum levels of miR-200c-3p have been found before in patients with metastatic CRPC as compared to localised PC or healthy controls [40, 41]. Similarly, miR-21-5p has been reported to be increased in plasma or serum of patients with PC as compared to healthy controls and patients with CRPC as compared to localised PC [40, 42, 43]. However, to the best of our knowledge, an association of EV-incorporated miR-200c-3p and miR-21-5p with PC has not been reported before. Hence, our study shows for the first time that EVs provide a better source for testing these miRNAs as PC biomarkers than whole plasma.

The only miRNA biomarker that could differentiate between PC patients with high vs low Gleason score was EV-incorporated Let-7a-5p, whose level was decreased in patients with Gleason score ≥ 8. This is in line with a study by Mihelich et al. showing that serum levels of Let-7a were decreased in PC patients with Gleason 4 + 5 grade tumours as compared with Gleason grade 3 [12]. Our study, though, shows that the whole plasma and EV levels of Let-7a-5p are poorly correlated and that EV-incorporated Let7a-5p level is more informative than Let7a-5p in whole plasma.

The cellular origin of circulating miRNAs is unclear. Although it seems likely that oncogenic miRNAs such as miR-375, miR-200c-3p and miR-21-5p that are overexpressed in PC tissues are released in the bloodstream from the tumour tissues, direct evidence for this is still lacking. On the contrary, Let-7a-5p is a tumour suppressive miRNA that is downregulated in PC tissues as compared to normal or BPH tissues [44, 45]. Hence, the decrease in Let-7a-5p plasma level in patients with aggressive PC is unlikely to be due to the release from cancer tissue. More plausibly, lower expression level or reduced release of Let-7a-5p is genetically associated with PC. Alternatively, it could be possible that signalling molecules produced by cancer cells actively downregulate the expression or release of this miRNA from normal tissues. In fact, a recent study by Chen et al. has demonstrated that breast cancer cells can downregulate the expression and release of miR-486 from cardiac and skeletal muscle in a TNFα-dependent manner [46], thus providing evidence that miRNAs released from non-tumour cells can have a diagnostic significance.

We did not observe diagnostic properties for circulating miR-30c-5p, miR-106a-5p, miR-141-3p, miR-223-3p and miR-210-3p in our patient cohort. The main reasons for this could be a relatively low sample size, the usage of different RNA isolation methods and different sample storage and processing conditions that may affect miRNA abundance and stability, and different normalisation methods for RT-qPCR results. In most of these studies, the results were normalised to the RNA input. Here, we normalised the RT-qPCR data against plasma volume and spike-ins that allow controlling for experimental variation. We reasoned that the quantity of EV-RNA in our samples is by far too small to be reliably measured by the currently available RNA quantification methods (e.g. Nanodrop, Qubit or Agilent Bioanalyzer), therefore the normalisation against RNA input may lead to biased results. Moreover, as EV levels have been found to be increased in cancer patients as compared to healthy controls [47], it seems likely that the levels of EV-enclosed RNA may also be increased, hence normalisation against the RNA input may result in the loss of diagnostically relevant information. Alternative approaches for RT-qPCR data normalisation include normalisation to an individual endogenous reference gene or the geometric mean of a set of normalisers. While the selection of housekeeping genes is relatively straight-forward for miRNA expression analysis in cells or tissues, the most commonly used internal control genes have turned out to be highly variable in biofluids [48–50], therefore, there is currently no consensus on appropriate normalisers in biofluids. Possibly, the most reliable normalisation strategy is a global geometric averaging of multiple genes, however this is applicable only when large panels of miRNAs are analysed. It should also be considered that EV-miRNA and cell-free miRNAs may require different normalization genes.

Furthermore, miR-141-3p and miR-106a-5p had discordant results across publications. Increased miR-106a serum levels were shown to correlate with increased CAPRA scores in one study [51], while another study showed that it is decreased in sera from PC patients with Gleason grades 4 + 5 as compared to grade 3 and BPH [12]. Increased serum or plasma levels of miR-141 have been found in PC patients as compared to healthy controls or BPH [1, 52], and in patients with metastatic CRPC as compared to localised PC [40, 53, 54]. At the same time, other studies reported that miR-141 was detectable in less than 50% of patients or had similar levels in PC patients and healthy controls [43, 54].

## Conclusions

To the best of our knowledge, this is the first study providing a head-to-head comparison of diagnostically relevant miRNA detection assays in whole plasma and plasma EVs from cancer patients. We show that only a minor fraction of the total cell-free miRNA could be recovered from the plasma EVs, however the EV-incorporated and whole plasma cell-free miRNA profiles were clearly different. Whole plasma MiR-375 could differentiate between PC and BPH, while miR-200c-3p and miR-21-5p performed better when analysed in EVs. EV-incorporated but not whole plasma Let-7a-5p level could distinguish patients with aggressive and indolent PC. This shows that EVs provide a more consistent source of RNA than whole plasma for the analysis of some miRNA biomarkers, while, possibly due to specific sorting mechanisms, the abundance of other miRNAs in EVs is very low and they show better diagnostic performance in whole plasma.

## Abbreviations

AUC: Area under the curve
BPH: Benign prostatic hyperplasia
CRPC: Castration resistant prostate cancer
EV: Extracellular vesicle
miRNA: MicroRNA
PSA: Prostate specific antigen
ROC: Receiver operator curve
